# Impact of Sex on Proper Use of Inhaler Devices in Asthma and COPD: A Systematic Review and Meta-Analysis

**DOI:** 10.3390/pharmaceutics14081565

**Published:** 2022-07-28

**Authors:** Luigino Calzetta, Marina Aiello, Annalisa Frizzelli, Beatrice Ludovica Ritondo, Elena Pistocchini, Paola Rogliani, Alfredo Chetta

**Affiliations:** 1 Respiratory Disease and Lung Function Unit, Department of Medicine and Surgery, University of Parma, 43126 Parma, Italy; marina.aiello@unipr.it (M.A.); annalisa.frizzelli@unipr.it (A.F.); alfredoantonio.chetta@unipr.it (A.C.); 2 Unit of Respiratory Medicine, Department of Experimental Medicine, University of Rome “Tor Vergata”, 00133 Rome, Italy; beatriceritondo@gmail.com (B.L.R.); elena.pistocchini@uniroma2.it (E.P.); paola.rogliani@uniroma2.it (P.R.)

**Keywords:** asthma, COPD, sex, meta-analysis, inhaler device, inhaler technique

## Abstract

Despite females being more often affected by asthma than males and the prevalence of COPD rising in females, conflicting evidence exists as to whether sex may modulate the correct inhaler technique. The aim of this study was to assess the impact of sex on the proper use of inhaler devices in asthma and COPD. A pairwise meta-analysis was performed on studies enrolling adult males and females with asthma or COPD and reporting data of patients making at least one error by inhaler device type (DPI, MDI, and SMI). The data of 6,571 patients with asthma or COPD were extracted from 12 studies. A moderate quality of evidence (GRADE +++) indicated that sex may influence the correct use of inhaler device in both asthma and COPD. The critical error rate was higher in females with asthma (OR 1.31, 95%CI 1.14–1.50) and COPD (OR 1.80, 95%CI 1.22–2.67) using DPI vs. males (*p* < 0.01). In addition, the use of SMI in COPD was associated with a greater rate of critical errors in females vs. males (OR 5.36, 95%CI 1.48–19.32; *p* < 0.05). No significant difference resulted for MDI. In conclusion, choosing the right inhaler device in agreement with sex may optimize the pharmacological treatment of asthma and COPD.

## 1. Introduction

Asthma and chronic obstructive pulmonary disease (COPD) are chronic respiratory diseases that affect over 600 million people worldwide and cause more than 3.5 million deaths annually [1,2,3].

Despite the availability of effective treatment options and well-established recommendations for disease diagnosis, management, and prevention [1,3], suboptimal symptom control and disease stability remain widespread among asthmatic and COPD patients [4]. Inadequate inhaler technique is one of the major determinants hampering proper disease control, and it has been associated with worse health status and a higher risk of hospitalization and acute exacerbations [5,6,7,8].

Inhaled medications are the gold standard for the treatment of asthma and COPD, having the advantage of rapid and high local drug delivery into the airways, while minimizing the risk of systemic side effects [9]. The Global Initiative for Asthma (GINA) [1] and the Global Initiative for Chronic Obstructive Lung Disease (GOLD) [3] recommendations recognized the importance of assessing patients’ inhaler technique regularly and providing inhaler skills training in case of poor inhalation technique before considering any step up in treatment.

A recent systematic review and meta-analysis by Chrystyn et al. [10] estimated that up to 100% of patients have made at least one overall error and up to 92% have made at least one critical error in handling inhaler devices, with a higher frequency of error rate reported in COPD patients compared to those with asthma. Device-specific handling errors in inhaler performance jeopardize the effectiveness of drug delivery, patient’s adherence to medication, and disease control, contributing substantially to health care costs [5,11].

Although errors in the proper use of inhaler devices have been addressed in terms of type of device [12,13] and patients’ preferences [13], limited and conflicting evidence exists as to whether sex may be a major predictor of inadequate inhaler performance [14,15]. In some studies, female sex was associated with an increased risk of errors in inhalation technique, with a higher proportion of men able to perform maneuvers correctly [14,16,17,18]. By contrast, other studies suggested a worse performance in males [19] or no real difference between females and males [20,21,22]. Even data originating from a meta-analysis did not result in conclusive findings [10].

Asthma morbidity is more common in female patients, and the prevalence of COPD is rising in females [23,24]. Moreover, raising attention has been drawn to the importance of examining sex-related differences in non-communicable diseases [25]. Thus, the implication that incorrect use of inhaler devices may be more common in females represents a major concern.

The current GINA [1] and GOLD [3] documents do not address the hypothesis that sex is associated with an incorrect use of inhaler devices in asthma and COPD. Thus, it may be assumed that the lack of any specific information could be due to there being no real difference between females and males in the ability of patients to use inhaler devices properly; conversely, no firm conclusion has yet been reached. Therefore, given such an uncertain background, the aim of the present systematic review and meta-analysis was to assess the impact of sex on the proper use of inhaler devices in asthma and COPD.

## 2. Materials and Methods

### 2.1. Search Strategy and Study Eligibility

**Figure 1:**
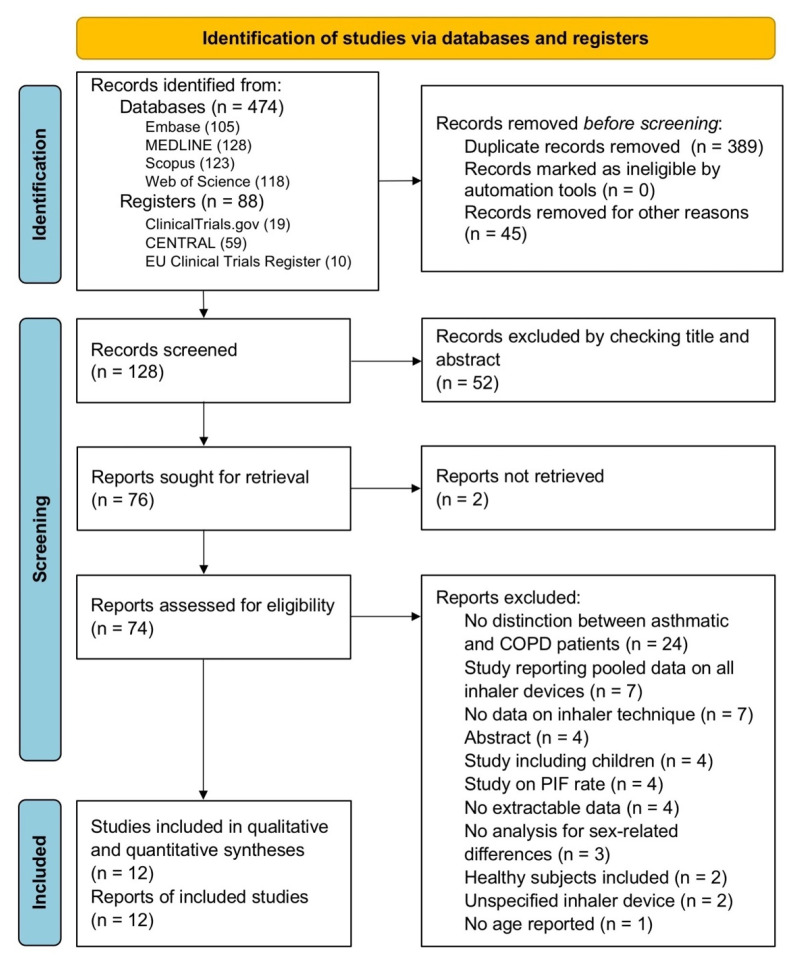
PRISMA 2020 flow diagram for the identification of the clinical studies included in the qualitative and quantitative syntheses. CENTRAL: Cochrane Central Register of Controlled Trials; PIF: peak inspiratory flow; PRISMA: Preferred Reporting Items for Systematic Reviews and Meta-Analyses.

The protocol of this systematic review and meta-analysis has been submitted to the international prospective register of systematic reviews (PROSPERO, registration ID: CRD42022333152) and performed in agreement with the Preferred Reporting Items for Systematic Reviews and Meta-Analyses Protocols (PRISMA-P) [26], and the relative flow diagram is shown in Figure 1. This study satisfied all the recommended items reported by the PRISMA 2020 checklist (Table S1) [27].

A comprehensive literature search was performed for clinical studies assessing the impact of sex on the proper use of inhaler devices for asthma or COPD. In this regard, the PICO (patient problem, intervention, comparison, and outcome) framework was applied to develop the literature search strategy, as previously reported [28]. Namely, the “patient problem” included patients suffering from asthma or COPD; the “intervention” regarded the use of different types of inhaler devices (dry powder inhaler (DPI), metered-dose inhaler (MDI), and soft mist inhaler (SMI)) for the administration of drugs in asthma or COPD; the “comparison” was performed between females and males; and the assessed “outcome” was the association between the improper use of inhaler devices and sex.

The search was performed in ClinicalTrials.gov, Cochrane Central Register of Controlled Trials (CENTRAL), Embase, EU Clinical Trials Register, MEDLINE, Scopus, and Web of Science, in order to provide relevant studies written in English and published from 1 January 1980 up to 3 January 2022. The summary of the search string was as follows: (sex OR gender) AND (asthma OR COPD). Detailed information regarding the expanded search string and translations are reported in Table S2. Citations of previous published reviews were checked to select further pertinent studies, if any [10,12,15,29,30].

Literature search results were uploaded to Eppi-Reviewer 4 (EPPI-Centre Software. London, UK), a web-based software program for managing and analyzing data in literature reviews that facilitates collaboration among reviewers during the study selection process.

### 2.2. Study Selection

Studies that enrolled adult males and females with asthma or COPD, written in English, and reporting the frequency or the number of patients making at least one overall error and/or an error exposing them to the risk of receiving a severely reduced dose or no medication being inhaled or reaching the lungs (referred to as critical error) [31] by specific inhaler device type were included in the meta-analysis.

Considering the limited knowledge regarding the impact of sex on inhaler technique [14,15], clinical trials, observational studies, and randomized controlled trial were selected in this meta-analysis in order to collect the greatest amount of data currently available on this topic. When the design of observational studies was not clearly reported, it was assessed by using previously published criteria [32].

The definition of critical error was in agreement with the International Pharmaceutical Aerosol Consortium on Regulation and Science (IPAC-RS) inhaler common use error matrix [33], developed for the assessment of use-related risk of harm caused by reduced dose delivery of a specific inhaler device. Critical error was considered equivalent to the IPAC-RS inhaler use error dosing scores of 7 and 10, corresponding respectively to the categories of high effect and maximal effect on the delivery to the lung of an individual dose [33].

Studies reporting combined data of a mixed population of patients with asthma and COPD or pooled data for all types of inhaler devices rather than for specific inhaler types (i.e., DPI, MDI, SMI) were not included in the analysis in order to perform a detailed analysis on specific diseases and devices and thus prevent the risk of affecting results by potential confounding factors.

Two reviewers independently examined the studies, and any difference in opinion concerning the selection of relevant studies from literature searches and databases was resolved by consensus.

### 2.3. Data Extraction

Data from included clinical studies were extracted from published papers and/or supplementary files. Data were checked for study references and characteristics and duration of observation, number of analyzed patients, type of inhaler device, patients’ diagnosis and setting, age, sex, forced expiratory volume in the 1st second (FEV_1_), FEV_1_/forced vital capacity (FVC), exacerbation ratio in the previous year, the frequency of overall errors, and/or critical errors by inhaler type, and study quality assessment via the Jadad Score [34], Cochrane Risk of Bias 2 (RoB 2) [35], and Joanna Briggs Institute (JBI) Critical Appraisal Checklist Tool [36].

Data regarding the proper use of inhaler devices were extracted from the primary publications as the number of patients making ≥1 error in inhaler technique, with no adjustments. When data were reported as odds ratio (OR), the number of patients making ≥1 error in inhaler technique was calculated from crude OR values.

Data were extracted in agreement with data extraction for complex meta-analysis (DECiMAL) recommendations [37]. The inter- and intra-rater reliability for data abstraction was assessed via the Cohen’s kappa score, as previously described [38]. Briefly, Cohen’s kappa ≥ 0.80 indicated excellent agreement, coefficients between 0.61 and 0.80 represented substantial agreement, coefficients between 0.41 and 0.61 moderate agreement, and <0.41 fair to poor agreement. 

### 2.4. Endpoint

The endpoint of this pairwise meta-analysis was to assess the impact of sex on the proper use of specific inhaler devices for the treatment of asthma and COPD.

### 2.5. Data Synthesis and Analysis

A pairwise meta-analysis was performed to assess the association between the frequency in making overall errors and/or critical errors by inhaler device type and sex. Results were expressed as OR and 95% confidence interval (95%CI). Since data were selected from a series of studies performed by researchers operating independently and a common effect size could not be assumed, a binary random-effects model was used to balance the study weights and adequately estimate the 95%CI of the mean distribution of the ORs for the investigated variables [39,40,41,42].

A subgroup analysis was conducted by grouping data from the overall analysis in agreement with the different types of inhaler devices.

The test for heterogeneity (I^2^) was performed to quantify the between-study dissimilarity, as previously reported [43]. Although a naïve ranking of values for I^2^ would not be standardized for all circumstances, low, moderate, and high heterogeneity may be identified for I^2^ values around 25%, 50%, and 75% [44]. Moreover, substantial heterogeneity may be considered for values of I^2^ greater than 50% [45]. Sensitivity analysis was carried out to identify the studies that introduced a significant and/or substantial level of heterogeneity (I^2^ > 50%) in the quantitative synthesis [44].

### 2.6. Quality of Studies and Risk Bias

The summary of the risk of bias for each included randomized controlled trial (RCT) was analyzed via the Cochrane RoB 2 [35] and Jadad score [34]. The weighted assessment of the overall risk of bias was analyzed via the Cochrane RoB 2 [35] by using the robvis visualization software [46,47].

The Jadad score, with a scale of 1–5 (a score of 5 being the best quality), was used to assess the quality of the RCTs concerning the likelihood of bias related with randomization, double blinding, withdrawals, and dropouts. The quality of studies was assessed as follows: total score ≤ 2, low quality; total score = 3, medium quality; total score ≥ 4 high quality.

The methodological quality of observational cross-sectional studies was evaluated by using the JBI Critical Appraisal Checklist Tool for analytical cross-sectional studies [36]. The checklist consisted of eight question items assessing the inclusion criteria for the definition and detailed description of the sample; use of valid and reliable way to measure the exposure; use of objective and standard criteria to measure the condition, identification, and strategies to deal with confounding factors; use of a valid and reliable way to measure outcomes; and suitability of statistical analysis. In the present systematic review and meta-analysis, each item of the JBI checklist was rated as “yes” and given 1 point or “no”, “unclear”, or “not applicable” and given 0 points. The quality assessment score was calculated on the proportion of “yes” responses for the possible maximum score and judges the results as high risk, moderate risk, or low risk of bias in agreement with the percentage of the achieved score, which was ≤49%, 50–69%, or ≥70%, respectively. 

The risk of publication bias was assessed by applying the funnel plot and Egger’s test if ≥10 studies were included in the meta-analysis [48] and in the case of a significant and/or substantial level of heterogeneity, as previously described [49]. 

The quality of the evidence was assessed in agreement with the Grading of Recommendations Assessment, Development, and Evaluation (GRADE) system, indicating ++++ for high quality of evidence, +++ for moderate quality of evidence, ++ for low quality of evidence, and + for very low quality of evidence [50].

Two reviewers independently assessed the quality of individual studies, and any difference in opinion about the quality score was resolved by consensus.

### 2.7. Software and Statistical Significance

GraphReader was used to extract data from the figures, when necessary (graphreader.com); Open-MetaAnalyst was used to perform the pairwise meta-analysis [43]; GRADEpro GDT to assess the quality of evidence [50]; and robvis visualization software to perform the RoB 2 tool [46,47]. The statistical significance was assessed for *p* < 0.05. 

## 3. Results

### 3.1. Study Characteristics

Of the 128 potentially relevant records identified in the initial search, 12 studies were deemed eligible for qualitative and quantitative syntheses. Data obtained from 5724 asthmatic patients and 847 COPD patients were extracted from 11 observational cross-sectional studies [51,52,53,54,55,56,57,58,59,60,61] and 1 RCT [31]. The study by Harb et al. [31] was interventional and randomized in design; however, the error frequency in inhaler technique was assessed at baseline, prior to verbal and demonstrative instructions on correct performance.

Of the overall 6571 patients, 3716 (56.55%) were females and 2855 (43.45%) were males. The relevant patient demographics, study characteristics, and study quality assessment have been summarized in Table 1. The investigated inhaler devices included DPI (Aerolizer, Accuhaler, Breezhaler, Diskhaler, Diskus, Easyhaler, Ellipta, Genuair, HandiHaler, Rotahaler, and Turbuhaler), MDI or pressurized MDI (pMDI) with or without a spacer (such as Ventolin, Atrovent, or Combivent), and SMI (Respimat). The inhaler technique was evaluated for DPI in 11 studies [31,51,53,54,55,56,57,58,59,60,61], for MDI in 8 studies [31,51,52,53,55,56,58,60], and for SMI in 2 studies [31,55]. Data on bronchoreversibility were not reported in Table 1 as none of the analyzed studies included this information.

The definition of critical error as reported in the studies is shown in Table 2. The inter-rater reliability for data abstraction was excellent before and after the learning process (Cohen’s Kappa 0.96 and 1.00, respectively). The intra-rater reliability produced a Cohen’s kappa of 1.00 after the learning process.

### 3.2. Pairwise Meta-Analysis

#### 3.2.1. Asthma

**Figure 2:**
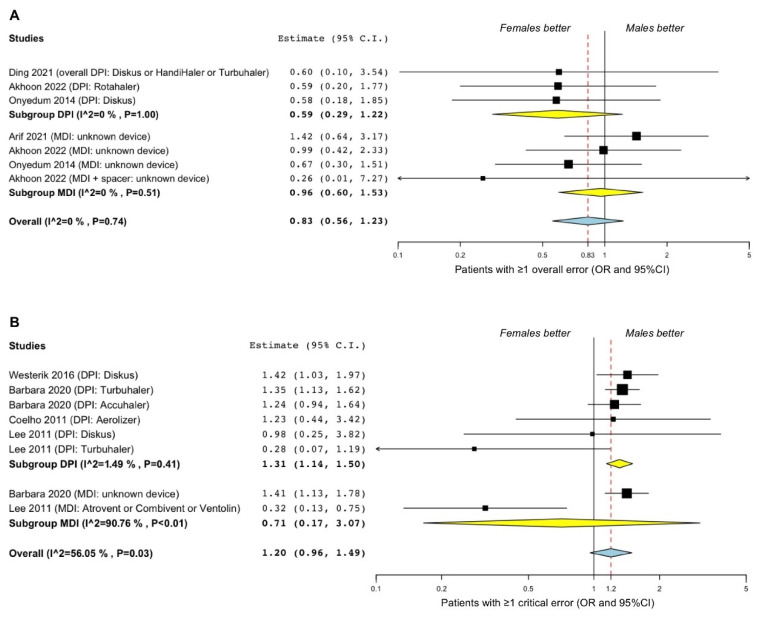
Forest plots of the association between the frequency of making at least one overall error (**A**) [51,52,54,60] or one critical error (**B**) [53,56,59,61] in the use of inhaler devices in asthma. DPI: dry powder inhaler; MDI: metered-dose inhaler; OR: odds ratio; 95%CI: 95% confidence interval.

No significant (*p* > 0.05) difference between females and males was observed in making at least one overall error in the use of inhaler devices (OR 0.83, 95%CI 0.56–1.23; I^2^ 0%; GRADE ++) (Figure 2A). The subgroup analysis confirmed the not significant (*p* > 0.05) association between sexes in the improper use of DPI and MDI, and no substantial heterogeneity was detected (Figure 2A).

There was a trend towards significance (*p* = 0.11) in the association of female sex with making at least one critical error in the use of inhaler devices (OR 1.20; 95%CI 0.96–1.49; I^2^ 56.05%; GRADE ++), which was mainly driven by the effect estimate resulting for DPI (Figure 2B). In this regard, the subgroup analysis indicated that significantly (*p* < 0.01) more female than male patients made at least one critical error in the use of DPI (OR 1.31, 95%CI 1.14–1.50; I^2^ 1.49%; GRADE +++), whereas no significant (*p* > 0.05) difference between sexes was detected for MDI (Figure 2B). The substantial and significant level of heterogeneity (I^2^ 90.76%, *p* < 0.01) detected in the subgroup analysis for MDI could not be resolved as only two studies [53,56] were included.

#### 3.2.2. COPD

**Figure 3:**
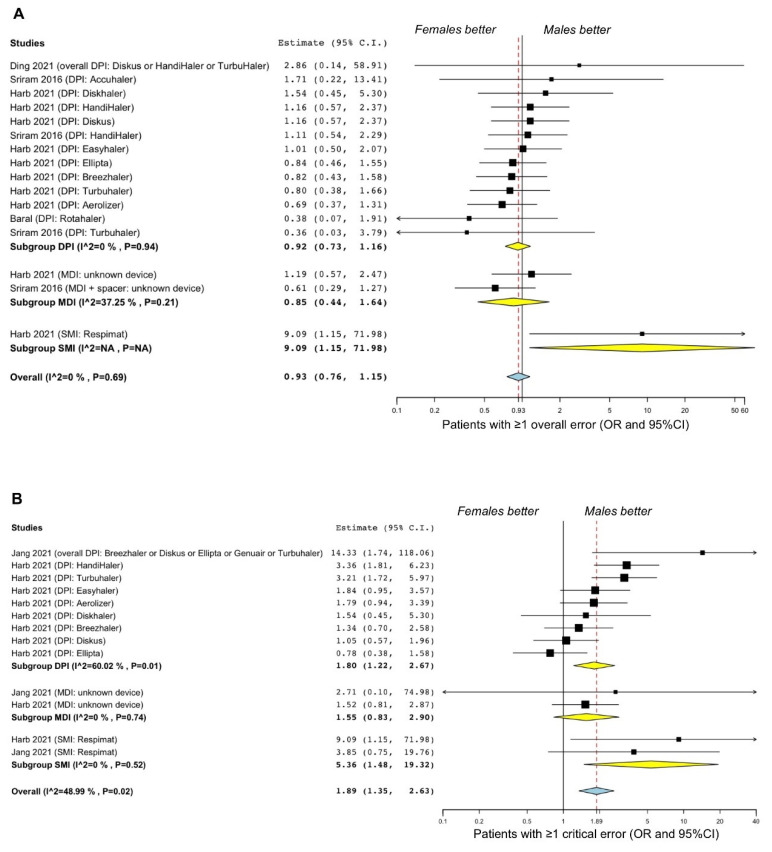
Forest plots of the association between the frequency of making at least one overall error (**A**) [31,54,57,58] or one critical error (**B**) [31,55] in the use of inhaler devices in COPD. DPI: dry powder inhaler; MDI: metered-dose inhaler; OR: odds ratio; SMI: soft mist inhaler; 95%CI: 95% confidence interval.

No significant (*p* > 0.05) difference was observed between females and males in making at least one overall error in the use of inhaler devices (OR 0.93, 95%CI 0.76–1.15; I^2^ 0%; GRADE ++) (Figure 3A). The subgroup analysis indicated no significant (*p* > 0.05) difference by sexes in the improper use of DPI and MDI, and no substantial heterogeneity was detected (Figure 3A). Female sex was significantly (*p* < 0.05) associated with making at least one overall error in the use of SMI (OR 9.09, 95%CI 1.15–71.98; GRADE +++), but the effect estimate resulted from only one study [31], and thus heterogeneity was not estimated (Figure 3A).

Sensitivity analysis performed by excluding the RCT of Harb et al. [31] in the DPI subgroup confirmed the not significant (*p* > 0.05) difference between sexes in making at least one overall error, and no substantial heterogeneity was detected (data not shown).

Patients making at least one critical error in the use of inhaler devices were significantly (*p* < 0.001) more likely to be female than male (OR 1.89, 95%CI 1.35–2.63; I^2^ 48.99%; GRADE +++) (Figure 3B). In the subgroup analysis, female sex was significantly (*p* < 0.01) associated with making at least one critical error in the use of DPI (OR 1.80, 95%CI 1.22–2.67; I^2^ 60.02%; GRADE +++) (Figure 3B). Sensitivity analysis indicated that the substantial and significant (*p* = 0.01) level of heterogeneity was resolved after removing from the analysis the study of Jang et al. [55] and that of Harb et al. [31] for the arm DPI Ellipta; the first study introduced a bias due to the small study effect [63], and the second one was the only study outlying on the left-hand side of the equality line. When significant and substantial heterogeneity was resolved by sensitivity analysis, results confirmed the significant (*p* < 0.001) association of female sex with making at least one critical error in the use of inhaler devices (OR 1.95, 95%CI 1.46–2.60; I^2^ 28.86; GRADE +++) and of DPI (OR 1.90, 95%CI 1.35–2.68; I^2^ 43.63%; GRADE +++).

Sensitivity analysis by excluding the RCT by Harb et al. [31] in the DPI subgroup could not be performed as this would have left only the study by Jang et al. [55] included, thus generating a single effect estimate.

No significant (*p* > 0.05) difference by sexes was observed in the improper use of MDI, and no substantial level of heterogeneity was detected (Figure 3B). Female sex was significantly (*p* < 0.05) associated with making at least one critical error in the use of SMI (OR 5.36, 95%CI 1.48–19.32; I^2^ 0%; GRADE ++) (Figure 3B).

### 3.3. Risk of Bias and Quality of Evidence

**Figure 4:**
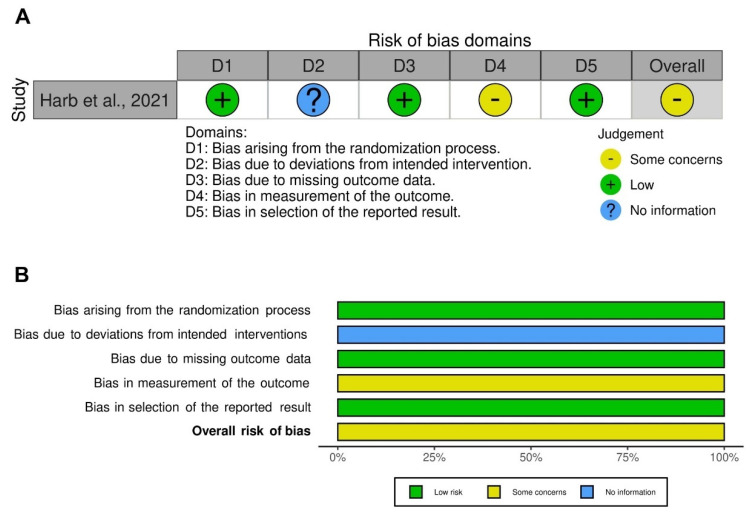
Assessment of the risk of bias via the Cochrane RoB 2 tool displayed by means of a traffic light plot of the risk of bias of the included randomized clinical study (**A**) [31], and weighted plot for the distribution of the overall risk of bias within each bias domain via the Cochrane RoB 2 tool (**B**) (*n* = 1 clinical study). Traffic light plot reports five risk of bias domains: D1, bias arising from the randomization process; D2, bias due to deviations from intended intervention; D3, bias due to missing outcome data; D4, bias in measurement of the outcome; D5, bias in selection of the reported result. Yellow circle indicates some concerns on the risk of bias, green circle represents low risk of bias, and blue circle indicates no information. RoB: risk of bias.

The RCT trial by Harb et al. [31] included in this pairwise meta-analysis was ranked as being of low quality (Jadad score < 3). The traffic light plot for the assessment of the risk of bias is reported in Figure 4A, and the weighted plot for the assessment of the overall risk of bias by domains is shown in Figure 4B. The RCT [31] had a low risk of bias for the randomization process, missing outcome data, and selection of the reported results. There were some concerns for the risk of bias in the measurement of the outcome and no information concerning deviations from intended intervention.

Quality assessment of observational cross-sectional studies indicated that 10 studies [51,52,54,55,56,57,58,59,60,61] were at moderate risk of bias and 1 study [53] was at high risk of bias.

Funnel plot and Egger’s test were not performed since less than 10 studies were included in the meta-analyses.

## 4. Discussion

The results of this systematic review and meta-analysis provide a moderate quality of evidence that sex may influence the correct use of inhaler device in asthma and COPD. The rates of critical error were greater in asthmatic and COPD female patients using DPI compared to male patients; furthermore, the use of SMI was associated with a significant greater overall and critical error rates in COPD female patients compared to male patients.

A previous meta-analysis [10] already investigated device errors in asthma and COPD, indicating sex, across the patients’ characteristics, as a factor that may have an impact on device error rates. Effectively, meta-regression suggested that females performed a higher error frequency [10]. However, that meta-analysis [10] was not specifically focused on inhaler device, and notably, results regarding improper inhaler technique were reported as pooled data from both asthmatic and COPD patients [17,64,65]. A recent overview [29] on interventions to improve inhaler technique also highlighted that female sex was associated with poorer technique, but again, the authors based their conclusions on primary studies performed on pooled populations of both asthmatic and COPD patients [17,64,65], or on studies in which no data were reported for a specific inhaler device (i.e., MDI, DPI, SMI) [18]. Therefore, to the best of our knowledge, this is the first study that systematically quantified the impact of sex on the proper use of specific inhaler devices in asthma and COPD.

Indeed, the obtained results may have important clinical implications. While male patients may correctly use any type of inhaler device, clinicians and nurses should carefully assess whether female patients are really able to correctly actuate a DPI for the treatment of asthma or either a DPI or a SMI for the treatment of COPD. In this respect, the incorrect use of inhaler device may lead to an increase in medication dose to reach disease control [10], an important matter in asthma management since the dose of medications is related to the step severity of the disease [1]. Moreover, in COPD, any critical error is a potential risk factor for frequent exacerbations [66]. Ultimately, especially for female patients, careful monitoring and education around inhaler devices are pivotal components of COPD treatments in frequent exacerbators and in poorly controlled asthmatics.

Although choosing the right inhaler device for the right patient is a substantial component of personalized and precision medicine [67,68], current real-life evidence suggests that prescription of a specific inhaler device is unrelated to the characteristics of asthmatic and COPD patients [69,70]. A recent extensive review [71] on the past, present, and future of inhaled medicines provided good evidence that the improper use of inhalers and incorrect mode of breathing may lead to overall non-adherence of around 50%. However, despite the large improvement in the engineering of inhaler devices, the authors [71] did not refer to sex as a key factor modifying the correct use of inhaler devices. Certainly, before investing in innovative “electronic”, “intelligent”, or “smart” inhalers [71], it could be more rational to optimize the correct use of the currently available devices already approved for the treatment of asthma and COPD, at least according to the sex of patients in agreement to the results of our quantitative synthesis.

It has been extensively demonstrated that inhaler errors may significantly affect drug delivery, explaining the significant association between improper inhaler device use and poor asthma control and COPD disease stability [15]. Thus, considering that most of the drugs and fixed-dose combinations (FDCs) currently approved for the treatment of asthma and COPD are delivered via both MDI and DPI, it should not be challenging for clinicians to prescribe the right device to female patients [72,73,74]. Concerning SMI, since the tiotropium bromide/olodaterol FDC is not delivered via other inhaler devices [72], female COPD patients should be adequately trained to correctly use this inhaler device if they must be treated specifically with tiotropium bromide/olodaterol FDC.

Generally, males are characterized by a better hand-to-eye coordination than females [75,76]. Thus, since inadequate hand-breath coordination may affect the proper use of MDI, along with poor fine motor control and hand or finger muscle weakness [77,78], we expected that females would have had more difficulty in using MDI instead of DPI. Conversely, we can suppose that the greater difficulty for females suffering from asthma and COPD in correctly using DPI may be related to a lower peak inspiratory flow (PIF) than male patients, as suboptimal PIF is a limiting factor for the correct actuation of DPI, a condition leading to inadequate drug-carrier disaggregation and insufficient drug deposition into the airways [79]. As a matter of fact, in both asthma and COPD, the PIF value reported for females is consistently lower than that reported for males, irrespective of the types of DPI used [80,81]. Effectively, previous findings suggested that female sex can be considered an independent risk factor for failing in the correct use of DPI in chronic obstructive airway disorders, mainly due to the differences in anthropometric features between the sexes affecting PIF [81,82]. Concerning the sex difference in the correct use of SMI, no previous findings are currently available; even a global systematic literature review and meta-analysis focused on device use errors with SMI did not investigate the potential impact of sex as a modifying factor [13]. Indeed, the slow-moving aerosol produced by SMI gives more time for a better inhalation-actuation coordination, which may enhance drug delivery [83]. However, according to the evidence that some patients may find the procedure of loading the cartridge into the inhaler challenging [79], we can speculate that sex difference in the development of three-dimensional spatial abilities [76] may explain the greater error rates reported for females compared to males in using SMI.

Certainly, this study has some limitations. First, we included in the quantitative synthesis both RCTs and observational studies; however, the sensitivity analysis performed by excluding the only RCT by Harb et al. [31] confirmed the effect estimate for the overall error rate. In any case, we have to highlight that integrating data from RCTs and observational studies in meta-analyses regarding complex interventions, such as the impact of sex on the correct use of inhaler devices, may improve the prediction of patient responses to therapies, regardless of the quality of included studies [84,85]. To date, there is no a priori reason to exclude observational studies from a qualitative synthesis [86,87], supporting that the greatest level in the new hierarchy of evidence is reached when both RCTs and observational studies exist with consistent findings [88]. Second, none of the studies included in the meta-analysis were specifically designed to assess the impact of sex on inhaler technique, explaining why a moderate risk of bias was generally reported by the JBI Checklist Tool. Only one study resulting from the PRISMA 2020 flow diagram evaluated the influence of sex on inhaler technique, but unfortunately, it reported data with no distinction between asthmatic and COPD patients, and for this reason the study was excluded at screening. Despite this limitation, the authors of [14] notably reported that patients reporting difficulties with using inhalers were more prevalent among females than males. Third, no consistent definition of “critical error” was stated; however, the several included studies [31,53,55,56,59,61] reported data for errors exposing a patient to the risk of receiving a severely reduced dose or no medication being inhaled or reaching the lungs, which can thus be referred to as critical errors, as shown in Table 2. Finally, the study by Jang et al. [55] was strongly underpowered to assess whether female sex could have been associated with the incorrect use of inhaler devices. In this regard, the sensitivity analysis confirmed that this study induced a bias via the so-called “small study effect”, a condition that leads to overrating of an effect estimate when it is assessed in small populations [89].

In conclusion, sex seems to be a significant factor modulating the correct use of inhaler device, with females performing more critical errors than men when using either DPI or SMI. Indeed, the quantitative synthesis of current evidence suggests that choosing the right inhaler device in agreement with sex may optimize the pharmacological treatment of asthma and COPD, or in other words, avoid that formulations are not properly inhaled. Further research is needed to improve the strength of the recommendations resulting from this study, by performing clinical trials specifically designed to assess the impact of sex on the proper use of inhaler devices in asthma and COPD.

## Figures and Tables

**Table 1:** Study characteristics of the clinical studies included in qualitative and quantitative syntheses.

Study, Year, and Reference	Study Characteristics	Observation Duration (Months)	Number of Analyzed Patients	Data Reported in the Primary Publication	Inhaler Device (Brand)	Patients’ Diagnosis (Setting)	Age (Years)	Male (%)	Post Bronchodilator FEV_1_ (% Predicted)	Post Bronchodilator FEV_1_/FVC	AECOPD in the Previous Year (Ratio)	JBI Checklist Tool	Evaluated Outcome
Akhoon et al., 2022 [51]	Single-center, observational, cross-sectional study	12.0	207	Number of patients making ≥1 overall error in inhaler technique	DPI (Rotahaler), pMDI (NA), and pMDI + spacer (NA)	Mild to moderate asthma (outpatient)	39.0	54.6	NA	NA	NA	Moderate bias	Patients making ≥1 overall error in inhaler technique
Arif et al., 2021 [52]	Single-center, observational, cross-sectional study	3.0	146	Crude OR with 95%CI for the association between sex and improper inhaler technique	MDI (NA)	Asthma (outpatient)	38.5	32.2	NA	NA	NA	Moderate bias	Patients making ≥1 overall error in inhaler technique
Ding et al., 2021 [54]	Single-center, observational, cross-sectional study	9.0	52 (COPD); 22 (asthma)	Number of patients making ≥1 overall error in inhaler technique	DPI (Diskus, HandiHaler, Turbuhaler)	Stable asthma, COPD (outpatient)	68.0 (COPD); 58.0 (asthma)	94.2 (COPD); 36.4 (asthma)	57.6 (COPD); 86.5 (asthma)	NA	NA	Moderate bias	Patients making ≥1 overall error in inhaler technique
Harb et al., 2021 [31]	Single-center, non-drug interventional, randomized, open-label, crossover study	NA	180	Number of patients making ≥1 overall error and ≥1 critical error in inhaler technique	DPI (Aerolizer, Breezhaler, Diskhaler, Diskus, Easyhaler, Ellipta, Handihaler, Turbuhaler), non-breath-actuated pMDI, and SMI (Respimat)	COPD (inpatient)	61.7	57.2	NA	NA	NA	NA *	Patients making ≥1 overall error and ≥1 critical error in inhaler technique
Jang et al., 2021 [55]	Single-center, observational, prospective, cross-sectional study (secondary analysis of a previous cohort study [62])	22.0	261	Number of patients making ≥1 critical error in inhaler technique	DPI (Breezhaler, Diskus, Ellipta, Genuair, Turbuhaler), pMDI, and SMI (Respimat)	COPD (outpatient)	69.8	93.5	63.5	0.59	24.9% of patients with frequent AECOPD	Moderate bias	Patients making ≥1 critical error in inhaler technique
Barbara et al., 2020 [56]	Multicenter, observational, retrospective cross-sectional study using data from the iHARP database	42.0	4,134	Number of patients making ≥1 critical error in inhaler technique	DPI (Accuhaler, Turbuhaler) and MDI (NA)	Asthma (primary care practice)	50.0	39.0	NA	NA	NA	Moderate bias	Patients making ≥1 critical error in inhaler technique
Baral et al., 2019 [57]	Single-center, observational, cross-sectional study	1.0	204	Number of patients making ≥1 overall error in inhaler technique	DPI (Rotahaler)	COPD (outpatient and inpatient)	67.2	46.1	<80.0	<0.7	NA	Moderate bias	Patients making ≥1 overall error in inhaler technique
Sriram et al., 2016 [58]	Single-center, observational, cross-sectional study	12.0	150	Number of patients making ≥1 overall error in inhaler technique	DPI (Accuhaler, HandiHaler, Turbuhaler) and MDI + spacer (NA)	COPD (inpatient and community-based participants)	70.3	52.0	NA	NA	1.7	Moderate bias	Patients making ≥1 overall error in inhaler technique
Westerik et al., 2016 [59]	Multicenter, observational, historical, cross-sectional study using data from the iHARP database	29.0	623	Number of patients making ≥1 critical error in inhaler technique	DPI (Diskus)	Asthma (primary care practice)	51.0	39.0	NA	NA	0.6	Moderate bias	Patients making ≥1 critical error in inhaler technique
Onyedum et al., 2014 [60]	Multicenter, observational, cross-sectional study	7.0	140	Number of patients making ≥1 overall error in inhaler technique	DPI (Diskus) and pMDI (NA)	Asthma (outpatient)	47.6	46.4	NA	NA	NA	High bias	Patients making ≥1 overall error in inhaler technique
Coelho et al., 2011 [61]	Single-center, observational, cross-sectional study	16.0	229	Number of patients making ≥1 critical error in inhaler technique	DPI (Aerolizer)	Severe asthma (outpatient)	≥18.0	21.4	NA	NA	NA	Moderate bias	Patients making ≥1 critical error in inhaler technique
Lee et al., 2011 [53]	Multicenter, observational, cross-sectional study	NA	223	Number of patients making ≥1 critical error in inhaler technique	DPI (Turbuhaler (Pulmicort and Symbicort), Diskus (Flixotide and Seretide)), and pMDI (Ventolin, Atrovent, or Combivent)	Asthma (outpatient)	56.7	50.4	NA	NA	NA	High bias	Patients making ≥1 critical error in inhaler technique

* Due to the interventional design of the study, the assessment of the risk of bias via JBI Checklist Tool was not performed. AECOPD: acute exacerbation of COPD; COPD: chronic obstructive pulmonary disease; DPI: dry powder inhaler; FEV_1_: forced expiratory volume in the 1st second; FVC: forced vital capacity; iHARP: Helping Asthma in Real People inhaler technique assessment initiative; JBI: Joanna Briggs Institute; MDI: metered-dose inhaler; NA: not available; pMDI: pressurized metered-dose inhaler; SMI: soft mist inhaler.

**Table 2:** Definitions of errors exposing patient to the risk of receiving a severely reduced dose or no medication being inhaled or reaching the lungs (referred to as critical errors), as reported in the included studies.

Author, Year, and Reference	Critical Error Definition
Harb et al., 2021 [31]	The definition of critical error agreed with the recently published IPAC-RS critical error matrix. The critical error was equivalent to IPAC-RS maximal effect (score 10) and IPAC-RS high effect (score 7); “critical errors presented within the checklist are those exposing patients to the risk of receiving no dose or severely reduced dose”.
Jang et al., 2021 [55]	“Critical errors were defined as errors seriously compromising drug delivery to the lung”.
Barbara et al., 2020 [56]	“Inhaler technique errors associated with poor asthma outcomes were defined as errors significantly associated with uncontrolled asthma and/or an increased rate of asthma exacerbations (ie, having at least one exacerbation in the 12 months prior to review)”.
Westerik et al., 2016 [59]	“Serious inhaler technique errors identified by the HCPs were defined as errors potentially limiting drug uptake to the lungs, as enumerated by the iHARP steering committee before commencing the study”.
Coelho et al., 2011 [61]	Error in a key step that “when incorrectly performed by users, can significantly affect total deposition of the dose in the lungs”. “These steps are related to preparing the dose for total drug release and to inhaling the drug”.The following were considered key steps in the present study: “for the use of an Aerolizer DPI, placing the capsule in the appropriate chamber, pressing the lateral buttons of the inhaler, and inhaling quickly and deeply”.
Lee et al., 2011 [53]	Failure of any one of the key steps, including “coordinate hand movement and inhalation,” “load and prime device,” and “inhale forcefully and deeply”.

DPI: dry powder inhaler; HCP: healthcare provider; iHARP: Helping Asthma in Real People inhaler technique assessment initiative; IPAC-RS: International Pharmaceutical Aerosol Consortium on Regulation & Science.

## Data Availability

The data presented in this study are available in the article.

## Supplementary Materials

The following supporting information can be downloaded at: https://www.mdpi.com/article/10.3390/pharmaceutics14081565/s1, Table S1: PRISMA 2020 Checklist; PRISMA: Preferred Reporting Items for Systematic Reviews and Meta-Analyses. Table S2: Expanded search string and translations.

## References

[1] GINA 2021 GINA Main Report|Global Initiative for Asthma. https://ginasthma.org/wp-content/uploads/2021/05/GINA-Main-Report-2021-V2-WMS.pdf.

[2] Asthma. https://www.who.int/news-room/fact-sheets/detail/asthma.

[3] GOLD (2022). Global Strategy for Prevention, Diagnosis and Management of COPD: 2022 Report. https://goldcopd.org/2022-gold-reports-2/.

[4] Roche N., Plaza V., Backer V., van der Palen J., Cerveri I., Gonzalez C., Safioti G., Scheepstra I., Patino O., Singh D. (2020). Asthma Control and COPD Symptom Burden in Patients Using Fixed-Dose Combination Inhalers (SPRINT Study). NPJ Prim. Care Respir. Med..

[5] Melani A.S., Bonavia M., Cilenti V., Cinti C., Lodi M., Martucci P., Serra M., Scichilone N., Sestini P., Aliani M. (2011). Inhaler Mishandling Remains Common in Real Life and Is Associated with Reduced Disease Control. Respir. Med..

[6] Lindgren S., Bake B., Larsson S. (1987). Clinical Consequences of Inadequate Inhalation Technique in Asthma Therapy. Eur. J. Respir. Dis..

[7] Virchow J.C., Crompton G.K., Dal Negro R., Pedersen S., Magnan A., Seidenberg J., Barnes P.J. (2008). Importance of Inhaler Devices in the Management of Airway Disease. Respir. Med..

[8] Vanoverschelde A., Van Der Wel P., Lahousse L. (2019). Poor Inhalation Technique Is a Major Determinant of Acute Exacerbations. Eur. Respir. J..

[9] Biddiscombe M.F., Usmani O.S. (2018). Is There Room for Further Innovation in Inhaled Therapy for Airways Disease?. Breathe.

[10] Chrystyn H., Van Der Palen J., Sharma R., Barnes N., Delafont B., Mahajan A., Thomas M. (2017). Device Errors in Asthma and COPD: Systematic Literature Review and Meta-Analysis. NPJ Prim. Care Respir. Med..

[11] Mäkelä M.J., Backer V., Hedegaard M., Larsson K. (2013). Adherence to Inhaled Therapies, Health Outcomes and Costs in Patients with Asthma and COPD. Respir. Med..

[12] Sanchis J., Gich I., Pedersen S. (2016). Systematic Review of Errors in Inhaler Use: Has Patient Technique Improved Over Time?. Chest.

[13] Navaie M., Dembek C., Cho-Reyes S., Yeh K., Celli B.R. (2020). Device Use Errors with Soft Mist Inhalers: A Global Systematic Literature Review and Meta-Analysis. Chronic Respir. Dis..

[14] Ocakli B., Ozmen I., Tuncay E.A., Gungor S., Ozalp A., Yasin Y., Adiguzel N., Gungor G., Karakurt Z. (2020). Influence of Gender on Inhaler Technique. Respir. Care.

[15] Usmani O.S., Lavorini F., Marshall J., Dunlop W.C.N., Heron L., Farrington E., Dekhuijzen R. (2018). Critical Inhaler Errors in Asthma and COPD: A Systematic Review of Impact on Health Outcomes. Respir. Res..

[16] Pothirat C., Chaiwong W., Phetsuk N., Pisalthanapuna S., Chetsadaphan N., Choomuang W. (2015). Evaluating Inhaler Use Technique in COPD Patients. Int. J. Chronic Obstr. Pulm. Dis..

[17] Goodman D.E., Israel E., Rosenberg M., Johnston R., Weiss S.T., Drazen J.M. (1994). The Influence of Age, Diagnosis, and Gender on Proper Use of Metered-Dose Inhalers. Am. J. Respir. Crit. Care Med..

[18] Duarte-De-Araújo A., Teixeira P., Hespanhol V., Correia-De-Sousa J. (2019). COPD: Misuse of Inhaler Devices in Clinical Practice. Int. J. Chronic Obstr. Pulm. Dis..

[19] Gray S.L., Williams D.M., Pulliam C.C., Sirgo M.A., Bishop A.L., Donohue J.F. (1996). Characteristics Predicting Incorrect Metered-Dose Inhaler Technique in Older Subjects. Arch. Intern. Med..

[20] Chafin C.C., Tolley E.A., George C.M., Demirkan K., Kuhl D.A., Pugazhenthi M., Self T.H. (2000). Gender Differences in Metered-Dose Inhaler-Spacer Device Technique. Pharmacotherapy.

[21] Arora P., Kumar L., Vohra V., Sarin R., Jaiswal A., Puri M.M., Rathee D., Chakraborty P. (2014). Evaluating the Technique of Using Inhalation Device in COPD and Bronchial Asthma Patients. Respir. Med..

[22] Giraud V., Roche N. (2002). Misuse of Corticosteroid Metered-Dose Inhaler Is Associated with Decreased Asthma Stability. Eur. Respir. J..

[23] Chowdhury N.U., Guntur V.P., Newcomb D.C., Wechsler M.E. (2021). Sex and Gender in Asthma. Eur. Respir. Rev..

[24] Calzetta L., Puxeddu E., Rogliani P. (2017). Gender-Related Responsiveness to the Pharmacological Treatment of COPD: A First Step Towards the Personalized Medicine. eBioMedicine.

[25] Mauvais-Jarvis F., Bairey Merz N., Barnes P.J., Brinton R.D., Carrero J.J., DeMeo D.L., De Vries G.J., Epperson C.N., Govindan R., Klein S.L. (2020). Sex and Gender: Modifiers of Health, Disease, and Medicine. Lancet.

[26] Moher D., Shamseer L., Clarke M., Ghersi D., Liberati A., Petticrew M., Shekelle P., Stewart L.A. (2015). Preferred Reporting Items for Systematic Review and Meta-Analysis Protocols (PRISMA-P) 2015 Statement. Syst. Rev..

[27] Page M.J., McKenzie J.E., Bossuyt P.M., Boutron I., Hoffmann T.C., Mulrow C.D., Shamseer L., Tetzlaff J.M., Akl E.A., Brennan S.E. (2021). The PRISMA 2020 Statement: An Updated Guideline for Reporting Systematic Reviews. BMJ.

[28] Schardt C., Adams M.B., Owens T., Keitz S., Fontelo P. (2007). Utilization of the PICO Framework to Improve Searching PubMed for Clinical Questions. BMC Med. Inf. Decis. Mak..

[29] Gleeson P.K., Feldman S., Apter A.J. (2020). Controller Inhalers: Overview of Devices, Instructions for Use, Errors, and Interventions to Improve Technique. J. Allergy Clin. Immunol. Pr..

[30] Biddiscombe M.F., Usmani O.S. (2021). Delivery and Adherence with Inhaled Therapy in Asthma. Minerva Med..

[31] Harb H.S., Ibrahim Laz N., Rabea H., Abdelrahim M.E.A. (2021). Determinants of Incorrect Inhaler Technique in Chronic Obstructive Pulmonary Disease Patients. Int. J. Clin. Pr..

[32] (2016). Understanding Observational Studies. Drug Ther. Bull..

[33] Stevens N., Dixon J., Lederhilger S., Mannerstråle F., Cheng J., Buckner C., Horst S., Limouzin K., Hoe L., Lyapustina S. (2020). The IPAC-RS Inhaler Common Use Errors Matrix.

[34] Jadad A.R., Moore R.A., Carroll D., Jenkinson C., Reynolds D.J., Gavaghan D.J., McQuay H.J. (1996). Assessing the Quality of Reports of Randomized Clinical Trials: Is Blinding Necessary?. Control. Clin. Trials.

[35] Higgins J.P.T., Savović J., Page M.J., Elbers R.G., Sterne J.A.C. (2019). Chapter 8: Assessing Risk of Bias in a Randomized Trial. Cochrane Handbook for Systematic Reviews of Interventions Version 6.0.

[36] Moola S., Munn Z., Tufanaru C., Aromataris E., Sears K., Sfetcu R., Currie M., Qureshi R., Mattis P., Lisy K., Aromataris E., Munn Z. (2020). Chapter 7: Systematic Reviews of Etiology and Risk. JBI Manual for Evidence Synthesis.

[37] Pedder H., Sarri G., Keeney E., Nunes V., Dias S. (2016). Data Extraction for Complex Meta-Analysis (DECiMAL) Guide. Syst. Rev..

[38] Gianinazzi M.E., Rueegg C.S., Zimmerman K., Kuehni C.E., Michel G., Swiss Paediatric Oncology Group (2015). Intra-Rater and Inter-Rater Reliability of a Medical Record Abstraction Study on Transition of Care after Childhood Cancer. PLoS ONE.

[39] Borenstein M. (2009). Introduction to Meta-Analysis.

[40] DeCoster J. (2004). Meta-Analysis Notes.

[41] Turner J.R., Durham T.A. (2014). Meta-methodology: Conducting and Reporting Meta-analyses. J. Clin. Hypertens..

[42] Cazzola M., Calzetta L., Page C., Jardim J., Chuchalin A.G., Rogliani P., Matera M.G. (2015). Influence of N-Acetylcysteine on Chronic Bronchitis or COPD Exacerbations: A Meta-Analysis. Eur. Respir. Rev..

[43] Wallace B.C., Dahabreh I.J., Trikalinos T.A., Lau J., Trow P., Schmid C.H. (2012). Closing the Gap between Methodologists and End-Users: R as a Computational Back-End. J. Stat. Softw..

[44] Higgins J.P.T., Thompson S.G., Deeks J.J., Altman D.G. (2003). Measuring Inconsistency in Meta-Analyses. BMJ.

[45] Higgins J.P., Green S. Cochrane Handbook for Systematic Reviews of Interventions. 9.5.2 Identifying and Measuring Heterogeneity. https://Handbook-5-1.Cochrane.Org/Chapter_9/9_5_2_identifying_and_measuring_heterogeneity.Htm.

[46] Sterne J.A.C., Savovic J., Page M.J., Elbers R.G., Blencowe N.S., Boutron I., Cates C.J., Cheng H.Y., Corbett M.S., Eldridge S.M. (2019). RoB 2: A Revised Tool for Assessing Risk of Bias in Randomised Trials. BMJ.

[47] McGuinness L.A., Higgins J.P.T. (2020). Risk-of-bias VISualization (robvis): An R package and shiny web app for visualizing risk-of-bias assessments. Res. Syn. Meth..

[48] Page M.J., Higgins J.P., Sterne J.A. (2019). Chapter 13: Assessing risk of bias due to missing results in a synthesis. Section 13.3.5.2 Funnel plots. Cochrane Handbook for Systematic Reviews of Interventions Version 6.0 (Updated July 2019).

[49] Calzetta L., Rogliani P., Matera M.G., Cazzola M. (2016). A Systematic Review with Meta-Analysis of Dual Bronchodilation with LAMA/LABA for the Treatment of Stable COPD. Chest.

[50] Guyatt G., Oxman A.D., Akl E.A., Kunz R., Vist G., Brozek J., Norris S., Falck-Ytter Y., Glasziou P., DeBeer H. (2011). GRADE Guidelines: 1. Introduction-GRADE Evidence Profiles and Summary of Findings Tables. J. Clin. Epidemiol..

[51] Akhoon N., Brashier D.B.S. (2022). A Study to Monitor Errors in Use of Inhalation Devices in Patients of Mild-to-Moderate Bronchial Asthma in a Tertiary Care Hospital in Eastern India. Perspect. Clin. Res..

[52] Arif N.B.M., Lee P.Y., Cheong A.T., Ananthan R.N.A. (2021). Factors Associated with Improper Metered-Dose Inhaler Technique among Adults with Asthma in a Primary Care Clinic in Malaysia. Malays. Fam. Physician.

[53] Lee S.M., Chang Y.S., Kim C.W., Kim T.B., Kim S.H., Kwon Y.E., Lee J.M., Lee S.K., Jeong J.W., Park J.W. (2011). Skills in Handling Turbuhaler, Diskus, and Pressurized Metered-Dose Inhaler in Korean Asthmatic Patients. Allergy Asthma Immunol. Res..

[54] Ding N., Zhang W., Wang Z., Bai C., He Q., Dong Y., Feng X., Zhang J., Gao S. (2021). Prevalence and Associated Factors of Suboptimal Daily Peak Inspiratory Flow and Technique Misuse of Dry Powder Inhalers in Outpatients with Stable Chronic Airway Diseases. Int. J. Chronic Obstr. Pulm. Dis..

[55] Jang J.G., Chung J.H., Shin K.C., Jin H.J., Lee K.H., Ahn J.H. (2021). Comparative Study of Inhaler Device Handling Technique and Risk Factors for Critical Inhaler Errors in Korean COPD Patients. Int. J. Chronic Obstr. Pulm. Dis..

[56] Barbara S.A., Kritikos V., Price D.B., Bosnic-Anticevich S. (2021). Identifying Patients at Risk of Poor Asthma Outcomes Associated with Making Inhaler Technique Errors. J. Asthma.

[57] Baral M.A. (2019). Knowledge and Practice of Dry Powder Inhalation among Patients with Chronic Obstructive Pulmonary Disease in a Regional Hospital, Nepal. Int. J. Gen. Med..

[58] Sriram K.B., Percival M. (2016). Suboptimal Inhaler Medication Adherence and Incorrect Technique Are Common among Chronic Obstructive Pulmonary Disease Patients. Chronic Respir. Dis..

[59] Westerik J.A.M., Carter V., Chrystyn H., Burden A., Thompson S.L., Ryan D., Gruffydd-Jones K., Haughney J., Roche N., Lavorini F. (2016). Characteristics of Patients Making Serious Inhaler Errors with a Dry Powder Inhaler and Association with Asthma-Related Events in a Primary Care Setting. J. Asthma.

[60] Onyedum C., Desalu O., Nwosu N., Chukwuka C., Ukwaja K., Ezeudo C. (2014). Evaluation of Inhaler Techniques among Asthma Patients Seen in Nigeria: An Observational Cross Sectional Study. Ann. Med. Health Sci. Res..

[61] Coelho A.C.C., Souza-Machado A., Leite M., Almeida P., Castro L., Cruz C.S., Stelmach R., Cruz Á.A. (2011). Use of Inhaler Devices and Asthma Control in Severe Asthma Patients at a Referral Center in the City of Salvador, Brazil. J. Bras. Pneumol..

[62] Ahn J.H., Chung J.H., Shin K.C., Jin H.J., Jang J.G., Lee M.S., Lee K.H. (2020). The Effects of Repeated Inhaler Device Handling Education in COPD Patients: A Prospective Cohort Study. Sci. Rep..

[63] Schwarzer G., Carpenter J.R., Rücker G. (2015). Small-Study Effects in Meta-Analysis.

[64] Chorão P., Pereira A.M., Fonseca J.A. (2014). Inhaler Devices in Asthma and COPD–An Assessment of Inhaler Technique and Patient Preferences. Respir. Med..

[65] Melani A.S., Zanchetta D., Barbato N., Sestini P., Cinti C., Canessa P.A., Aiolfi S., Neri M. (2004). Inhalation Technique and Variables Associated with Misuse of Conventional Metered-Dose Inhalers and Newer Dry Powder Inhalers in Experienced Adults. Ann. Allergy Asthma Immunol..

[66] Ahn J.H., Chung J.H., Shin K.C., Choi E.Y., Jin H.J., Lee M.S., Nam M.J., Lee K.H. (2019). Critical Inhaler Handling Error Is an Independent Risk Factor for Frequent Exacerbations of Chronic Obstructive Pulmonary Disease: Interim Results of a Single Center Prospective Study. Int. J. Chronic Obstr. Pulm. Dis..

[67] Canonica G.W., Ferrando M., Baiardini I., Puggioni F., Racca F., Passalacqua G., Heffler E. (2018). Asthma: Personalized and Precision Medicine. Curr. Opin. Allergy Clin. Immunol..

[68] Bakakos P., Chatziapostolou P., Katerelos P., Efstathopoulos P., Korkontzelou A., Katsaounou P. (2022). Extrafine Beclometasone Dipropionate/Formoterol NEXThaler on Device Usability, Adherence, Asthma Control and Quality of Life. A Panhellenic Prospective, Non-Interventional Observational Study in Patients with Asthma-The NEXT-Step Study. J. Pers. Med..

[69] Lavorini F., Bianco A., Blasi F., Braido F., Corsico A.G., Di Marco F., Gentile A., Paggiaro P.L., Pegoraro V., Pelaia G. (2020). What Drives Inhaler Prescription for Asthma Patients? Results from a Real-Life Retrospective Analysis. Respir. Med..

[70] Molimard M., Colthorpe P. (2015). Inhaler Devices for Chronic Obstructive Pulmonary Disease: Insights from Patients and Healthcare Practitioners. J. Aerosol Med. Pulm. Drug Deliv..

[71] Anderson S., Atkins P., Bäckman P., Cipolla D., Clark A., Daviskas E., Disse B., Entcheva-Dimitrov P., Fuller R., Gonda I. (2022). Inhaled Medicines: Past, Present, and Future. Pharmacol. Rev..

[72] Rogliani P., Calzetta L., Coppola A., Cavalli F., Ora J., Puxeddu E., Matera M.G., Cazzola M. (2017). Optimizing Drug Delivery in COPD: The Role of Inhaler Devices. Respir. Med..

[73] Scichilone N. (2015). Asthma Control: The Right Inhaler for the Right Patient. Adv. Ther..

[74] Lavorini F., Janson C., Braido F., Stratelis G., Løkke A. (2019). What to Consider before Prescribing Inhaled Medications: A Pragmatic Approach for Evaluating the Current Inhaler Landscape. Ther. Adv. Respir. Dis..

[75] Ali A., Subhi Y., Ringsted C., Konge L. (2015). Gender Differences in the Acquisition of Surgical Skills: A Systematic Review. Surg. Endosc..

[76] Jones C.M., Braithwaite V.A., Healy S.D. (2003). The Evolution of Sex Differences in Spatial Ability. Behav. Neurosci..

[77] Yawn B.P., Colice G.L., Hodder R. (2012). Practical Aspects of Inhaler Use in the Management of Chronic Obstructive Pulmonary Disease in the Primary Care Setting. Int. J. Chronic Obstr. Pulm. Dis..

[78] Bartolo K., Balzan M., Schembri E.L., Asciak R., Mercieca Balbi D., Pace Bardon M., Montefort S. (2017). Predictors of Correct Technique in Patients Using Pressurized Metered Dose Inhalers. BMC Pulm. Med..

[79] Ohar J.A., Ferguson G.T., Mahler D.A., Drummond M.B., Dhand R., Pleasants R.A., Anzueto A., Halpin D.M.G., Price D.B., Drescher G.S. (2022). Measuring Peak Inspiratory Flow in Patients with Chronic Obstructive Pulmonary Disease. Int. J. Chronic Obstr. Pulm. Dis..

[80] Altman P., Wehbe L., Dederichs J., Guerin T., Ament B., Moronta M.C., Pino A.V., Goyal P. (2018). Comparison of Peak Inspiratory Flow Rate via the Breezhaler^®^, Ellipta^®^ and HandiHaler^®^ Dry Powder Inhalers in Patients with Moderate to Very Severe COPD: A Randomized Cross-over Trial. BMC Pulm. Med..

[81] Haughney J., Lee A.J., McKnight E., Pertsovskaya I., O’Driscoll M., Usmani O.S. (2021). Peak Inspiratory Flow Measured at Different Inhaler Resistances in Patients with Asthma. J. Allergy Clin. Immunol. Pr..

[82] Taylor T.E., Holmes M.S., Sulaiman I., Costello R.W., Reilly R.B. Influences of Gender and Anthropometric Features on Inspiratory Inhaler Acoustics and Peak Inspiratory Flow Rate. Proceedings of the Annual International Conference of the IEEE Engineering in Medicine and Biology Society, EMBS.

[83] Dalby R.N., Eicher J., Zierenberg B. (2011). Development of Respimat^®^ Soft Mist^TM^ Inhaler and Its Clinical Utility in Respiratory Disorders. Med. Devices: Evid. Res..

[84] Alexander J., Edwards R.A., Savoldelli A., Manca L., Grugni R., Emir B., Whalen E., Watt S., Brodsky M., Parsons B. (2017). Integrating Data from Randomized Controlled Trials and Observational Studies to Predict the Response to Pregabalin in Patients with Painful Diabetic Peripheral Neuropathy. BMC Med. Res. Methodol..

[85] Arditi C., Burnand B., Peytremann-Bridevaux I. (2016). Adding Non-Randomised Studies to a Cochrane Review Brings Complementary Information for Healthcare Stakeholders: An Augmented Systematic Review and Meta-Analysis. BMC Health Serv. Res..

[86] Norris S., Atkins D., Bruening W., Fox S., Johnson E., Kane R., Morton S.C., Oremus M., Ospina M., Randhawa G. (2008). Selecting Observational Studies for Comparing Medical Interventions. Methods Guide for Effectiveness and Comparative Effectiveness Reviews.

[87] Shrier I., Boivin J.F., Steele R.J., Platt R.W., Furlan A., Kakuma R., Brophy J., Rossignol M. (2007). Should Meta-Analyses of Interventions Include Observational Studies in Addition to Randomized Controlled Trials? A Critical Examination of Underlying Principles. Am. J. Epidemiol..

[88] Gershon A.S., Jafarzadeh S.R., Wilson K.C., Walkey A.J. (2018). Clinical Knowledge from Observational Studies: Everything You Wanted to Know but Were Afraid to Ask. Am. J. Respir. Crit. Care Med..

[89] Calzetta L., Aiello M., Frizzelli A., Bertorelli G., Rogliani P., Chetta A. (2021). Oral Corticosteroids Dependence and Biologic Drugs in Severe Asthma: Myths or Facts? A Systematic Review of Real-World Evidence. Int. J. Mol. Sci..

